# Is Dentistry a Dangerous Profession

**Published:** 1891-10

**Authors:** Dudley W. Buxton

**Affiliations:** Member of the Royal College of Physicians.


AMERICAN JOURNAL

—OF—

DENTAL SCIENCE.

VOL. XXV. BALTIMORE, OCTOBER, 1891. NO. 6.

ARTICLE I.

### IS DENTISTRY A DANGEROUS PROFESSION.

BY DUDLEY W. BUXTON, M. D., LOND., B. S.

Member of the Royal College of Physicians.

Some few years back Mr. Oakley Coles, then a leading member of the dental profession, brought before the Odontological Society of Great Britain the subject comprehended by the question which stands as a caption to the present arttricle. It may appear an unnecessary, nay even a frivolous, theme upon which to occupy the valuable hours of our already overburdened lives, but I venture to think that a few moments of thought will enable us to show the question is no mere mental gymnastic, and that in its answer is involved the happiness or misery of many, while to discuss what is a natural pendant to our theme, the the hygiene of the dentist's life involves matters of the utmost importance to the busy brethren who form the dental profession. It has been alike my duty and my privilege to work with and watch at the bedside of not a few dentists and dental students, and the knowledge thereby gained has set my mind athinking again and again upon the problems how should a dentist live so as to gain health and retain it, in what manner may he obtain the greatest amount of work with the least amount of wear and tear.

It must I think be conceded that the life is a hard one, not so much from the length of time during which the individual is called upon to work as on account of the physical sameness, the unequal distribution of muscular strain and the tension involved in conscientious discharge of the duties comprising the diurnal life of the ordinary dentist. Again the work is all indoors and is carried on in more or less tainted air. Over and above these drawbacks to the calling there are those which involve his nervous system, congestive neuritis from standing in one position, the strain to the eyes and the nerve tension incident to due coordination of eye and hand involved in the accomplishment of fine and delicate manipulative dexterity. Probably no profession takes quite the same position as regards its effects upon health as dentistry, and I venture to think none offers quite so many opportunities for noxious influences to attack the individual. Luckily, however, that even with this proneness to run off the rail of health, the life of the dentist need not be really unwholesome. There are pitfalls it is true, and these are numerous as well as dangerous, but Nature kindly gives us excellent warning notes of danger, and we possess knowledge adequate to grapple with the troubles which arise, and still better, enables us being forewarned to be forearmed by calling in the assistance of prophylactic methods and relying upon the plain teachings of hygiene.

It would be folly to attempt to lay down hard and fast rules for men who although following the same calling yet differ so widely alike in the exigencies of their circumstances, in their tastes, and in their physical equipment. To suggest various plans for lessening the evils may subserve a useful purpose, while the adaptation of the rules themselves to suit variety of circumstances must be the work of the individual. What has struck me has been the prevalence of neurasthenia, nervous irritability, diseases clearly traceable to impaired nourishment of the nerve centres, and peripheral nerves showing themselves in neuralgias, *e. g.*, migraine, sciatica, herpes, zoster, asomnia, etc., etc.; all evidences it will be said of chronic overwork, but when we find them occurring in persons whose hours of labor are not unduly prolonged, we are compelled to believe that the nature of the work rather than its quantity is responsible for its bringing about these untoward results.

To secure immunity from neurasthenic troubles, *i. e.*, from affections or diseases, the result of nerve exhaustion, certain precautions should be taken, and these we propose to consider. (1.) number of hours of continuous labor. (2.) change of work. (3.) clothing, (4.) arrangement of day. (5.) hygiene of work room. (6.) recreation.

In considering the first heading, it is a very difficult matter to be more than vague, and therefore unsatisfactory. Some men can take appointments from 9 a. m. or even 8.30 until 6 or even 7 at night, trusting to the "slack time" for easying off, and do not, to the casual observer, appear to suffer. That they do themselves an injury eventually I have no doubt, and I believe, were we able to get at reliable life insurance official statistics we should find that the expectancy of life of such persons is certainly lessened. However, the number of dentists is too few to base any conclusions upon insurance figures, the more so as I find, that as a profession, they are not provident in the sense of insuring at all adequately, when it is considered that they make relatively large salaries or incomes early in life, and so marry young. Another flaw in this way of getting at the truth about the longevity of dentists is that the affections noted above are perhaps actually less inimical to life than to health, most tend, that is, to bring about rather a condition of chronic invalidism, than to determine premature decease. Providence against sickness, involving the principle of paying yearly premiums to secure a certain weekly allowance during partial or complete disablement through illness has not until within the past few years taken any hold upon the medical profession, and although the various offices now undertake the underwriting of these health risks, there is no special one for dentists as there is for members of the British Medical Association. The Benevolent Fund, that wise provision of the corresponding Association of Dentists works, I believe, upon eleemosynary lines, and therefore, of course, does not take the place of an insurance association against sickness, injury or death. If an engineer, or constant traveller should insure against accident, certainly the dentist should against sickness.

The average health limit of continuous work should be fixed at six hours a day, the hours of labor over this limit are of inferior quality, *as a rule*, of course many exceptions occur. A senior student of Cambridge, who subsequently came out senior wrangler, was asked by a "ten-hour man" how it was he read for "only five hours a day," and his reply, that although he read five hours only, yet while he read, he *read*, applies with equal force to other matters besides reading for examinations. There is a great deal of very indifferent work done by professional men, not perhaps done dishonestly, but nevertheless scamped work, and this is the direct result of an attempt to get through more than the tired hand and unnerved brain are capable of duly carrying out. Hard workers will readily agree that it is futile for them to look forward to "slack times" for recuperation, for, like a falling body, the hard worker gains momentum, as he travels along, the habit grows, and when the slack time arrives, a hundred and one thing intrude themselves into the day's routine, and transform its anticipated leisure into as great a stress of work as usual, for these additional things which have to be done must be deposed of before the "busy time" again comes round. Thus it happens that unless the hard worker makes a determined stand, and reduces by scale the hours in which professional work is to be done, he never gets free from, and as year succeeds year, he grows more than ever the slave of his profession. Hence the absolute importance of restricting the hours of labor. Then comes the question of income. It certainly is a fallacy to say that given a limit set to work, an equivalent amount must be written off the earnings. What has been shown in the great labor movements applies to professional men. In a factory where overtime was habitually paid, and men labored for 11 to 14 hours daily, an eight hours limit was expertimentally tried, and the wages fixed at a rate which was equivalent to those given for the long day's labor plus the overtime. The employer did this expecting to lose heavily, but to his surprise he found the men actually got through more and better work during the 8 than they had hitherto done when slaving on through 14 hours. So the professional man will, I venture to think, find that both the quality and quantity of his work will really be increased, if he limits his hours. To insure this, however, there must of course be time-saving appliances, appointments must be kept alike by patient and dentist, and when possible, the hall attendant or secretary must see that all preliminaries are duly attended to before the patient enters the operating room. Patients soon learn to respect punctuality, and the more readily apprehend its importance if sins of omission are made to involve an equivalent loss of time allotted to them while their fees are still exacted. Here again, a most important point is that the monetary arrangements should, when possible, be taken out of the hands of the principal. In this regard, it may be said, that for young men commencing practice, it is childish to suggest the keeping of a secretary, or official collection of fees and professional charges, nor do I deny that it is so, but such junior men have the leisure for such administrative work, and should carry it out, and only hand it over to others when their time becomes sufficiently valuable to demand outside help. This point is, I submit, reached, when six hours a day *good* work is required at their hands. All details of work, such as having instruments arranged orderly and at hand, bottles duly filled and properly arranged, everything in its right place, and that right place known, and all apparatus, water, electric, etc., in perfect order are presupposed, for the man who muddles his surgery will certainly muddle his work, and will as indubitably lose more time than he uses.

When we speak of change of work or variety of occupation, it is assumed that the cares and duties of the profession are not allowed to monopolize all the working day. Recreative work, provided it does not tax the same senses in the same way as do the routine duties of life, is certainly a benefit to the individual, and can moreover be made to indirectly assist his life's work by cultivating his eye, or his touch, etc. Thus the pursuits of art, painting, etching, photography, undoubtedly assist indirectly the dentist, just as a few hours at the engineer's bench, or with the lathe, strengthen while they give him manipulative dexterity. Even in change of work there is a danger, for unless due care be exercised the point of physiological equilibrium is overstepped, and instead of the "off" work being recreative it becomes exhausting, and so, detrimental.

Upon the matter of clothing, much stress was laid when Mr. Oakley Coles' paper was read upon the necessity for warm and absorptive undervesture, and there is an undoubted need of this. The equalizing of temperature which the skin has to perform is assisted by the use of Jaeger, or some such system of clothing, but there is another and none the less important point, which is, that a complete change of costume be made after the day's work. The operating room should be kept at a lower temperature than ordinary dwelling rooms, and hence the dress of the dentist should be warmer than his usual habiliments, and of course warmer than his outdoor dress.* The working dress should be loose, the feet and wrists and throat should be well protected, and straps or tight bands or badly designed braces carefully avoided, as in a constrained position bands become tight, which are loose enough when the body is held erect. Great care should be used to avold bad habits as regards standing, *e. g.*, cramping postures, stooping, and especially permitting the weight of the body to rest for long upon one leg. The dentist's stool of course obviates much standing, but very many dentists find its use more irksome than standing. The ambidextrous have a decided advantage in being able to alternate their work.

The arrangement of the day is no unimportant detail. Whatever may be the hour fixed for starting work it should be preceded by a good breakfast and walk, or better still ride either upon a horse or a cycle. Gentle exercise is what is required, anything beyond this means unsteadying of the hands, and many persons find cycling in the early morning causes too much fatigue and tremulousness of the hands. When this is so, walking for a mile or so should be adopted instead of it. An early rise, a cup of milk, and a piece of bread and butter, then the walk to be followed by a bath and breakfast is the best routine. Letters can then be glanced at and arranged to be dealt with subsequently. The day's work begun should not be continued for more than four hours without a break. The midday interval allows for a light luncheon, many find a biscuit enough, but when a more substantial lunch, *e.g.*, fish or fowl with rice, or other farinaceous pudding can be taken without inducing sleepiness or languor, it is better than the lighter repast, since that leads to an excessive dinner, which taken at 7 p. m. or 8 p. m., entails a severe tax upon the digestive functions. It is a common, but most objectionable practice for persons who have no appointment to call

---

* In outdoor exercise, whether walking, or riding, or cycling, the clothing if absorptive need not be very warm.

during this mid-day hour, upon the chance of "catching" the professional man, this should be ruthlessly checked. The professional day should close about 5 p. m., and steady exercise in the open air must be taken, whether wet or fine, and the dinner taken not later than 8 p. m., although 7 p. m. is a better time, as if the rational rule is followed of retiring to bed at 11 p. m., three good hours are thus afforded for digestion. Eight hours sleep is a proper allowance, and unless something like this be taken, asomnia and mental irritability are liable to show themselves. The physical fatigue of the day's work often leads dentists to forego walking and betake themselves tc driving. As a matter of fact, this is a mistake, the fatigue felt is really not fatigue, but is rather exhaustion of brain, coupled perhaps with a little straining of one set of muscles. If this felling is combated and the walk persisted in, there will follow in most cases a reward, and that is, the fatigue rapidly wear off and exhilaration succeed. The nearest high ground and open space should be selected for the walk.

Upon the subject of the hygiene of the surgery, very much might be written. The best light comes from a north or north-eastern aspect, glare is a danger to be carefully avoided, especially when refractive ocular troubles exist, such as astigmation, but while too strong an illumination should be avoided, on account of the fatigue incident, under these circumstances to the retina, especially a dim light should be avoided, as it engenders ocular disease by strain, often revealing itself in headache and visual vertigo. Ventilation should occupy an important place in the arrangement of a surgery. Ventilation, not draught, is the desideratum, and the periodic introduction of a small hurricane into the room, by throwing open doors and windows, is a mistake and a dangerous nuisance. A fire-place and an open window guarded by a board, placed in its plane to prevent a direct inrush of air, constitute a simple and effectual system of ventilation, when the more elaborate system of Tobin's tubes are not available. Unshutable sources of ventilation are preferable to others, as most persons have a morbid dread of fresh air, and will, if they can, get a room stuffy. But flap windows falling from above, and even revolving window-pane plates have their uses. Heavy carpets and much furniture, especially if carved, are agreeable to the æsthetic sense, but are out of place in a hygienic surgery, as the most conscientious cleaner cannot keep the place free from dust and dirt from carious teeth, and lung exhalation is not devoid of danger to those who spend many hours in its neighborhood. Painted walls, polished floors, and matting capable of daily or at least frequent wiping down, with a minimum of stuffed furniture, and an exclusion of saddle-bag settees constitute, I venture to think, a good general outline of what we should find in a dentist's room. Books, heavily framed pictures, shields, etc., should not be allowed. The light need hardly now be dwelt on, for most persons fitting up their houses would employ electricity both for light and motor. In other cases, gas may be used, but only after consultation with a gas engineer, for although gas may be feeble as an illuminator, hurtful as a calorifier, and harmful from a health point of view, it need be none of them. With care it may afford a splendid light, be made an efficient means of ventilating, and be excluded from the respirable air of the room. The Wenham system, when the rooms are large enough is a good one. Uncovered gas jets are bad in economy, deleterious to the eyes and the health. Open fire-places afford the best, although perhaps the most costly mode of warming, many gas stoves are good, but they need careful working, and it is difficult not to get a used up condition of the air. Sprays of some terebinthine compound such as sanitas, terebene, pumiline, or better still the occasional burning of a few logs of Scotch pine, give an agreeable odor and ozonise the air of the room. The large amount of putrescent material which must circulate in the atmosphere of the dentist's room, and find its way into his own lungs as he inhales the breath of his patients should make him most careful to adopt such precautions. Bacteriology teaches us practical lessons, and one seems to me to be that the dentist's work brings him into constant contact with those mischiefs which nature yet leaves for idle germs to do.

Many operators adopt a very bad habit of holding, or half-holding their breath while operating this is decidedly injurious, as it diminishes the air supply to the lungs, and interferes with due depuration of the pulmonary air.

Much stress must also be laid upon the importance to dentists of gymnastic exercises. "I am no athlete," may be said, but no one is too old or too stiff to use dumb-bells and light clubs. These must, however, be skilfully handled, and graduated exercises learnt or schemed to open the thorax, and ensure a daily thorough wash-out of the pulmonary vascular system, and so obviate stagnating pulmonary apices.

When we turn to consider recreation, we find much again to say, but space compels me to curtail my remarks. I would preach moderation in all things, lest the fate which befell the literary lady befalls the more energetic of dentists. Writing with enthusiasm for far longer hours than her brain could properly accomplish, she took her leisure in hoeing potatoes and digging, and in these pursu ts her energy again hurried her into excesses, resulting in a paralytic disease, which in time crippled alike her mind and her body. Out-door pursuits of course stand first, air and exercise, cycling, riding, or when the weather is perverse, rackets, and failing these, a good walk and a Turkish bath stand pre-eminent for exercise. Mental recreation, I will not venture to insist upon, as in these days of culture, everyone reads, and some few think, while yet fewer go first hand to Nature for her enchanting teaching. The same hedge-rows which spoke to poor Richard Jeffreys are yet eloquent for those who have ears to hear; the sunsets which inspired Turner are still occurring day-byday; while in London, and the other great cities, Nature is not wholly stamped out, but offers food for thought, work for the microscope, the pencil and the brush, and our great cities also afford opportunities for hearing such music as may well make one rejoice in living. What more need I say? Possibly it will be replied by the cynic that enthusiasm on the matter of music, and of Nature's teaching is out of date; if it be so, I trust someone will have the courage to put back the hands of the clock, that we may all again grow as enthusiastic in our play as in our work, for after all, enthusiasm only means putting one's whole energy into the matter, and without energy, the spice of life has gone, and the work becomes slave's toil, while the play is a sorry travesty of mirth.—*London Dental Record.*

---

ARTICLE II.

